# Sublethal UV irradiation induces squamous differentiation via a p53-independent, DNA damage-mitosis checkpoint

**DOI:** 10.1038/s41419-018-1130-8

**Published:** 2018-10-25

**Authors:** Isabel de Pedro, Pilar Alonso-Lecue, Natalia Sanz-Gómez, Ana Freije, Alberto Gandarillas

**Affiliations:** 1 Cell Cycle, Stem Cell Fate and Cancer Laboratory, Marqués de Valdecilla Research Institute (IDIVAL), 39011 Santander, Spain; 2 INSERM, Languedoc-Roussillon, 34394 Montpellier, France

## Abstract

The epidermis is a self-renewal epithelium continuously exposed to the genotoxic effects of ultraviolet (UV) light, the main cause of skin cancer. Therefore, it needs robust self-protective mechanisms facing genomic damage. p53 has been shown to mediate apoptosis in sunburn cells of the epidermis. However, epidermal cells daily receive sublethal mutagenic doses of UV and massive apoptosis would be deleterious. We have recently unravelled an anti-oncogenic keratinocyte DNA damage-differentiation response to cell cycle stress. We now have studied this response to high or moderate single doses of UV irradiation. Whereas, as expected, high levels of UV induced p53-dependent apoptosis, moderate levels triggered squamous differentiation. UV-induced differentiation was not mediated by endogenous p53. Overexpression of the mitosis global regulator FOXM1 alleviated the proliferative loss caused by UV. Conversely, knocking-down the mitotic checkpoint protein Wee1 drove UV-induced differentiation into apoptosis. Therefore, the results indicate that mitosis checkpoints determine the response to UV irradiation. The differentiation response was also found in cells of head and neck epithelia thus uncovering a common regulation in squamous tissues upon chronic exposure to mutagens, with implications into homeostasis and disease.

## Introduction

Stratified epithelia of the skin and head and neck are continuously exposed to mutagenic carcinogens. Skin cancer in all forms (melanoma and carcinoma) has strikingly increased  in the last decades due to social trends such as tanning or outdoor sports. It is well established that the main causes of epithelial skin cancer are continuous exposure to the genotoxic effect of ultraviolet (UV) and continuous cell renewal^1–4^. Skin sunburn has been found to trigger apoptosis of severely damaged keratinocytes in the epidermis^5,6^. However, sublethal chronic UV irradiation impacts the keratinocyte genome even in the absence of burning and this is the main cause of precancerous mutations. Induction of massive apoptosis in the epidermis upon UV irradiation would compromise the skin function. The fate of moderately non-lethal UV-damaged keratinocytes and the mechanisms by which the epidermis avoids its precancerous potential are unclear.

Tumour suppressor p53 is very frequently mutated in skin carcinomas in a UV-traceable and specific manner^4,5^. p53 is referred to as the guardian of the genome due to its important role in controlling the cell cycle and inducing apoptosis upon DNA damage^7^. Healthy sun-exposed skin contains patches of cells displaying mutant p53 although a relationship with skin cancer has not been found^8–10^. The fate of these mutant cells is uncertain.

We have previously revealed a keratinocyte DNA damage-differentiation response (DDDR) to cell cycle deregulation or mitotic inhibition^11,12^. Interestingly, knock-down of p53 or overexpression of proto-oncogene MYC or the cell cycle promoter Cyclin E in primary cells via replication stress^13^ triggers the DDDR and results in squamous cell differentiation and shedding. This response is controlled by a differentiation-mitosis checkpoint (DMC)^14^.

Since UV irradiation causes DNA damage and G2/M arrest^15–17^, we have investigated whether sublethal levels trigger the DMC. The results show that, as expected, acute high levels of UV irradiation in human primary keratinocytes cause apoptosis mediated by p53. However, more moderate levels of UV irradiation that were sublethal yet significantly causing DNA damage, induced mitotic arrest and terminal differentiation. Contrary to UV-induced apoptosis, this response was independent of p53. Interestingly, UV-induced differentiation was attenuated by forcing mitosis when overexpressing FOXM1. In addition, we provide evidence for a role of a Wee1-mediated mitotic checkpoint in the differentiation response. The results provide new insight into the mechanisms limiting the clinical impact of cell sublethal UV irradiation in skin. They contribute explaining why UV irradiation is therapeutic on the psoriatic skin or why chronic or persistent irradiation is needed for skin carcinomas to develop. The observation that oral keratinocytes also differentiate terminally in response to UV irradiation to which they are not usually exposed, suggests common mechanisms of squamous epithelia facing genetic damage.

## Results

To determine the DNA damage caused by UV light in human keratinocytes, we performed a dose-response study. As shown in Supplementary Figure 1, all doses tested produced a significant increase in the DNA damage marker γH2AX 5 h after irradiation as measured by flow cytometry and immunofluorescence. Most cells were positive for the marker at any dose. However, 300 mJ/cm^2^ caused a stronger induction of the marker than 25 mJ/cm^2^ (intensity level 2, Supplementary Figure 1). An early fraction of apoptotic cells was detected at the higher doses but not at the lower doses (25 mJ/cm^2^; sub-G1; Supplementary Figure 1a). As expected, there was an induction of the tumour suppressor p53 upon UV irradiation (green; Supplementary Figure 1b). In response to DNA damage, p53 holds the cycle to allow DNA repair^7^.

**Fig. 1:**
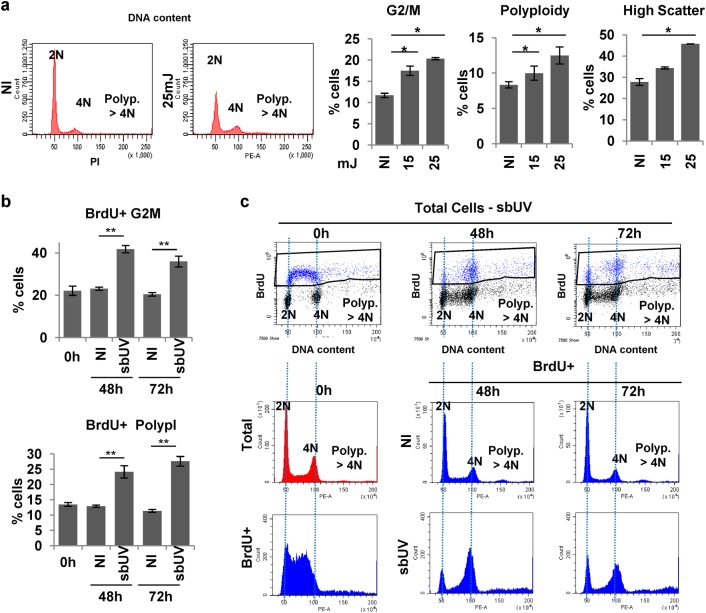
Sublethal UV irradiation induces DNA synthesis in epidermal keratinocytes. Primary human epidermal keratinocytes 48 h (**a–c**) or 72 h (**b**, **c**) after sublethal UV irradiation (15/25 mJ/cm^2^ as indicated). **a** Representative flow-cytometry analyses of the cell cycle (DNA content by Propidium Iodide; PI). Bar histograms: percent of cells in the G2/M (4N) or polyploid (>4N) fractions of the cell cycle or displaying high light scattering (forward vs side scatter), as indicated. **b** Percent of BrdU positive cells in the G2/M (upper panel) or the polyploid (polypl; lower panel) fractions of the cell cycle. **c** Representative flow cytometry analyses of BrdU pulse-chase experiments to follow the positive cells after irradiation (positive cells in blue). Cells were labelled for 1.5 h just before irradiation (0 h) and harvested at the time-points indicated (0, 48, 72 h). Upper panels: dot plots for DNA content (X axes) vs BrdU (Y axes, positive cells in blue). Lower panels: DNA content profiles (PI) for total or BrdU positive cells as indicated. NI: non-irradiated. Irradiation units: /cm^2^. *p < 0.05, **p < 0.01. Data are representative or mean ± s.e.m. of triplicate samples. See also Supplementary Figure 2 and 3

We aimed to determine the keratinocyte fate after doses of UV irradiation that were sublethal (sbUV). In order to investigate this, we chose doses 15–25 mJ/cm^2^. First, we analysed the effect on the cell cycle. As shown in Supplementary Figure 2a (DNA content), only a small proportion of cells was found in sub-G1, indicative of apoptosis, even 72 h after irradiation. At these doses, UV produced a decrease in the G1 phase of the cell cycle and an increase in the G2/M phases (4N) and polyploidy (>4N; Fig. 1a and Supplementary Figure 2a). Similar results were observed upon 15 or 25 mJ/cm^2^ with slight variation in the timing of the response, the latter causing a moderately faster response. Either dose of irradiation provoked a significant increase in the cellular light scatter parameters of cells, reflecting a switch to higher size and complexity typical of keratinocyte differentiation (Fig. 1a and Supplementary Figure 2a^18^). Up-regulation of p53 by the effect of sbUV irradiation was sustained (Supplementary Figure 2b).

UV light is thought to activate the cell cycle^19,20^. The cell cycle profiles that we observed were consistent with this model. However, the accumulation of cells in S phase and G2/M could be due to cells being stuck due to DNA breaks not to cell cycle activation. Therefore, we performed BrdU incorporation assays upon sbUV irradiation to measure the proportion of cells undergoing DNA replication. DNA synthesis was significantly increased 12 h after irradiation (Supplementary Figure 3a). Consistent with a mitosis arrest, irradiated DNA synthesising cells also accumulated in G2/M. In order to monitor DNA re-replication beyond G2/M after irradiation, we performed pulse-chase experiments. Keratinocytes were labelled for 1.5 h just before irradiation. Interestingly, BrdU positive accumulated mainly in G2/M by 48 h and progressed into polyploidy by 72 h (Fig. 1b, c and Supplementary Figure 3b), demonstrating that cells continued DNA replication in spite of a mitotic block.

**Fig. 2:**
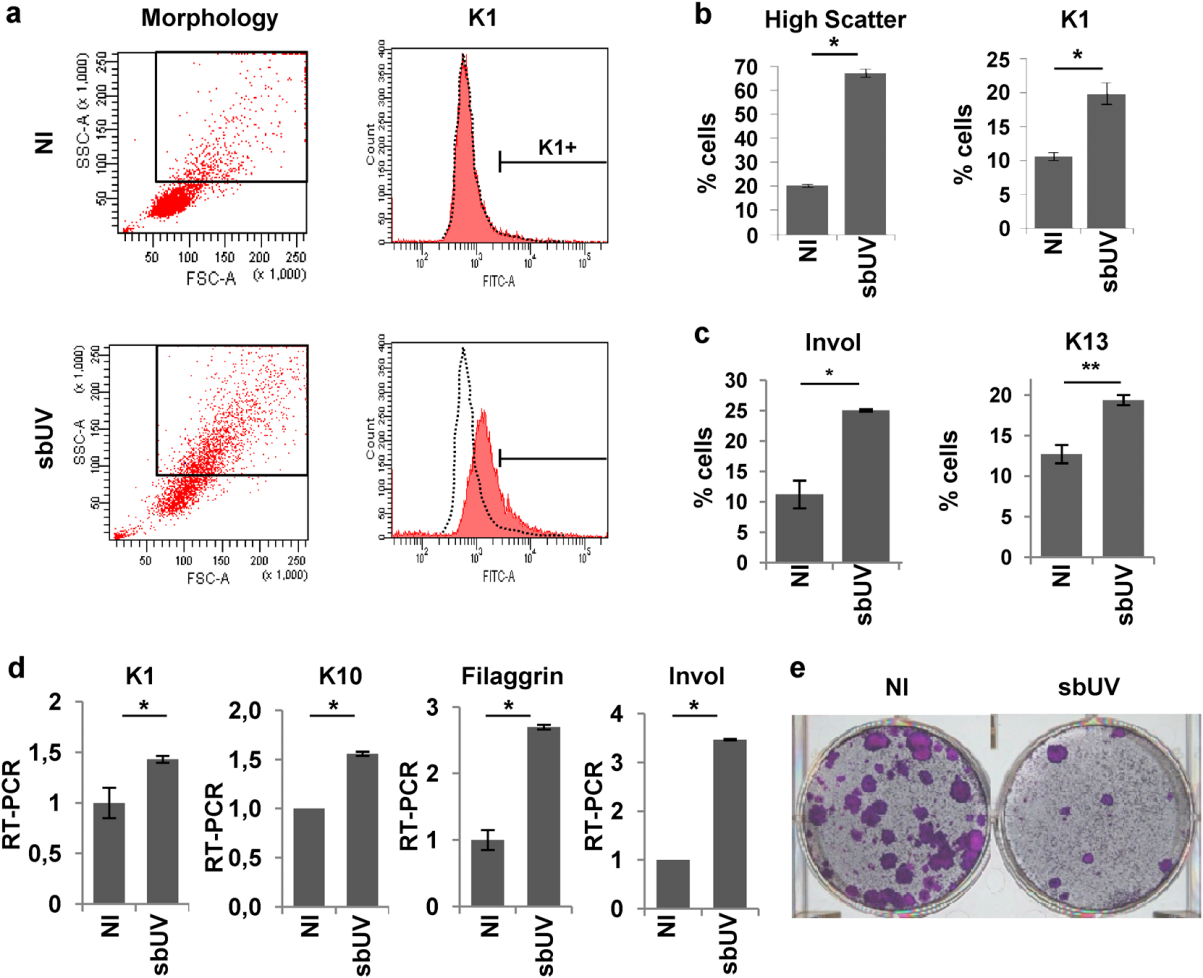
Sublethal UV irradiation induces squamous differentiation in epidermal keratinocytes. Primary human epidermal keratinocytes 24 h (**d**) or 48 h (**a**–**c**, **e**) after sublethal UV irradiation (sbUV; 15 mJ/cm^2^). **a** Representative flow-cytometry analyses for morphology (light scatter) or the expression of keratin K1. Black boxes gate cells with high light scatter typical of differentiation. **b** Percent of high scatter cells or keratin K1 positive cells. **c** Percent of involucrin (Invol) or keratin K13 positive cells. **d** Expression of K1, K10, filaggrin or Invol as measured by RT-qPCR. **e** Clonogenic capacity of cells plated 48 h after UV irradiation as in (**a**) (3000 cells per well in triplicates). NI: non-irradiated. Irradiation units: /cm^2^. *p < 0.05, **p < 0.01. Data are representative or mean ± s.e.m. of triplicate samples. See also Supplementary Figure 4

Consistent with the cell cycle changes and the increase in light scattering, sbUV irradiation (15/25 mJ/cm^2^) induced the expression of epidermal suprabasal protein markers keratins K1, K13 and K16 and involucrin (Fig. 2a–c and Supplementary Figure 4a-c). RT-PCR also demonstrated an increase in the expression of markers keratins K1 and K10, filaggrin and involucrin (Fig. 2d). Further evidence that sbUV irradiation induced terminal differentiation, was the differentiated morphology of irradiated cells (Supplementary Figure 4d) and the striking irreversible loss of proliferative potential (Fig. 2e and Supplementary Figure 4e).

**Fig. 3:**
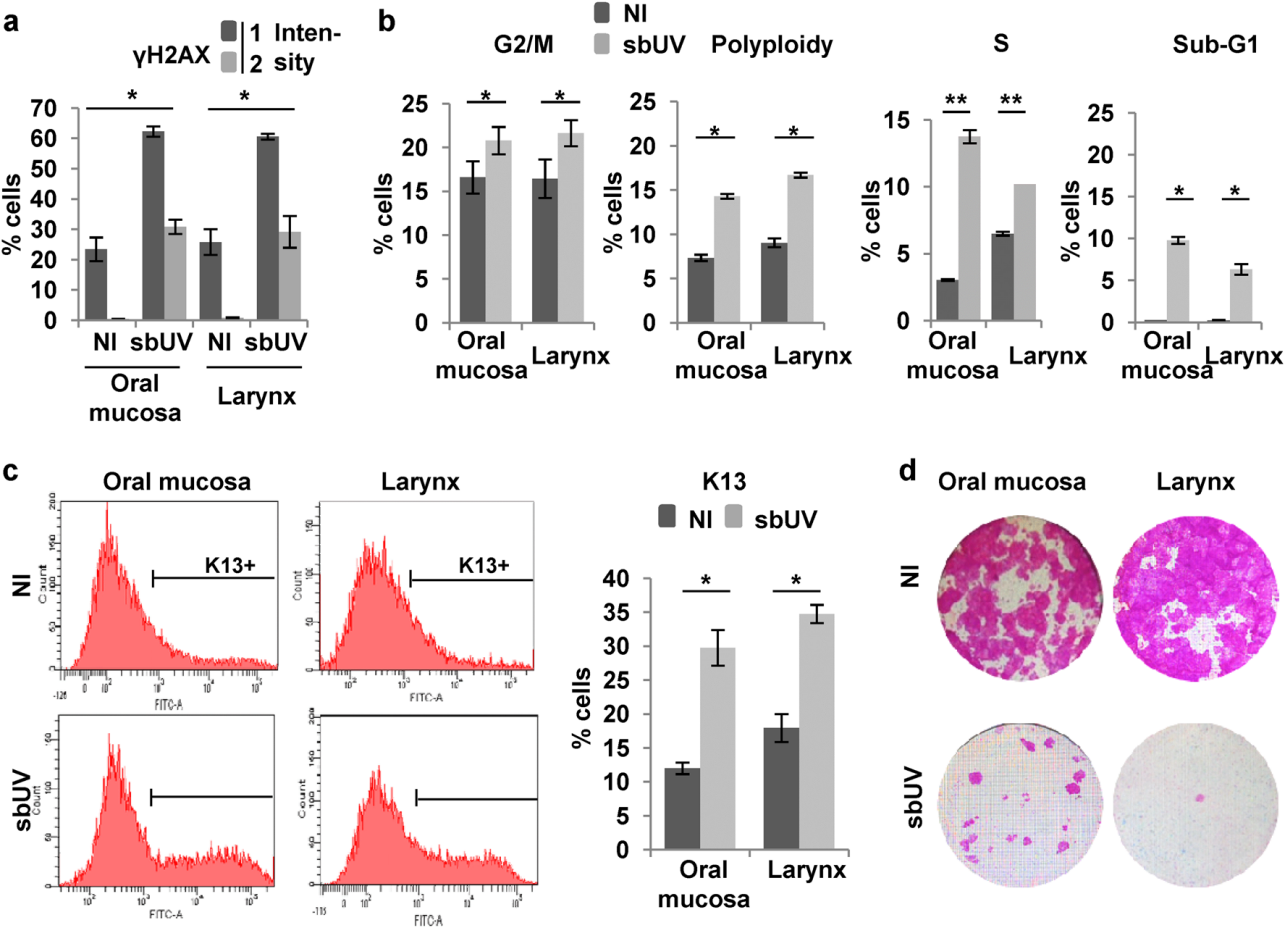
Sublethal UV irradiation induces squamous differentiation in keratinocytes of head and neck. Primary cells from human oral mucosa or larynx after sublethal UV irradiation (sbUV; 25 mJ/cm^2^) were analysed 5 h (**a**) or 48 h (**b**–**d**) after irradiation. **a** Percent of γH2AX (early DNA damage marker) positive cells according to 2 levels of intensity (1–2). **b** Percent of G2/M, polyploid, S or sub-G1 fractions of the cell cycle, measured as in Fig. 1. **c** Representative flow cytometry analyses of the differentiation marker keratin K13. Bar histogram shows the percent of K13 positive cells (K13+) as determined by a negative control antibody (see Materials and methods) **d** Clonogenic capacity of cells plated 48 h after irradiation (2500 cells per well in triplicates). NI: non-irradiated. *p < 0.05, **p < 0.01. Data are representative or mean ± s.e.m. of duplicate samples and two independent experiments. See also Supplementary Figure 5

We have recently found that stratified epithelia of head and neck share with epidermal keratinocytes a DDDR to cell cycle deregulation^21^. We aimed to test whether head and neck squamous cells respond similarly to UV irradiation even though they are not usually exposed to this carcinogen. Therefore, we isolated primary cells from oral mucosa and larynx and subjected them to sbUV irradiation. As shown in Fig. 3, these cells responded in a similar way as epidermal keratinocytes. Irradiated oral and larynx cells greatly expressed the DNA damage marker γH2AX and accumulated in S phase, G2/M and polyploidy (Fig. 3a, b and Supplementary Figure 5). A low fraction of sub-G1 cells was observed in the cell cycle profiles at these doses (9% oral and 6% larynx; Fig. 3b and Supplementary Figure 5b). The irradiation induced the expression of the squamous differentiation marker keratin K13 (Fig. 3c). Consistent with the induction of irreversible terminal differentiation, the clonogenic capacity of these cells was lost upon UV irradiation (Fig. 3d).

The loss of clonogenicity in irradiated keratinocytes would not be due to quiescence as cells were more actively on cycle. The induction of all markers of postmitotic differentiation suggests that cells ceased proliferation due to terminal differentiation. However, we tested whether some of the keratinocyte proliferative potential lost upon sbUV irradiation was due to senescence. We analysed cells for Beta-Galactosidase activity (B-Gal), the main marker of this process^22^. While mitomycin C-treated fibroblasts proved positive for B-Gal, none of the keratinocyte colonies were positive, before or even 12 days after sbUV irradiation (Supplementary Figure 6).

**Fig. 4:**
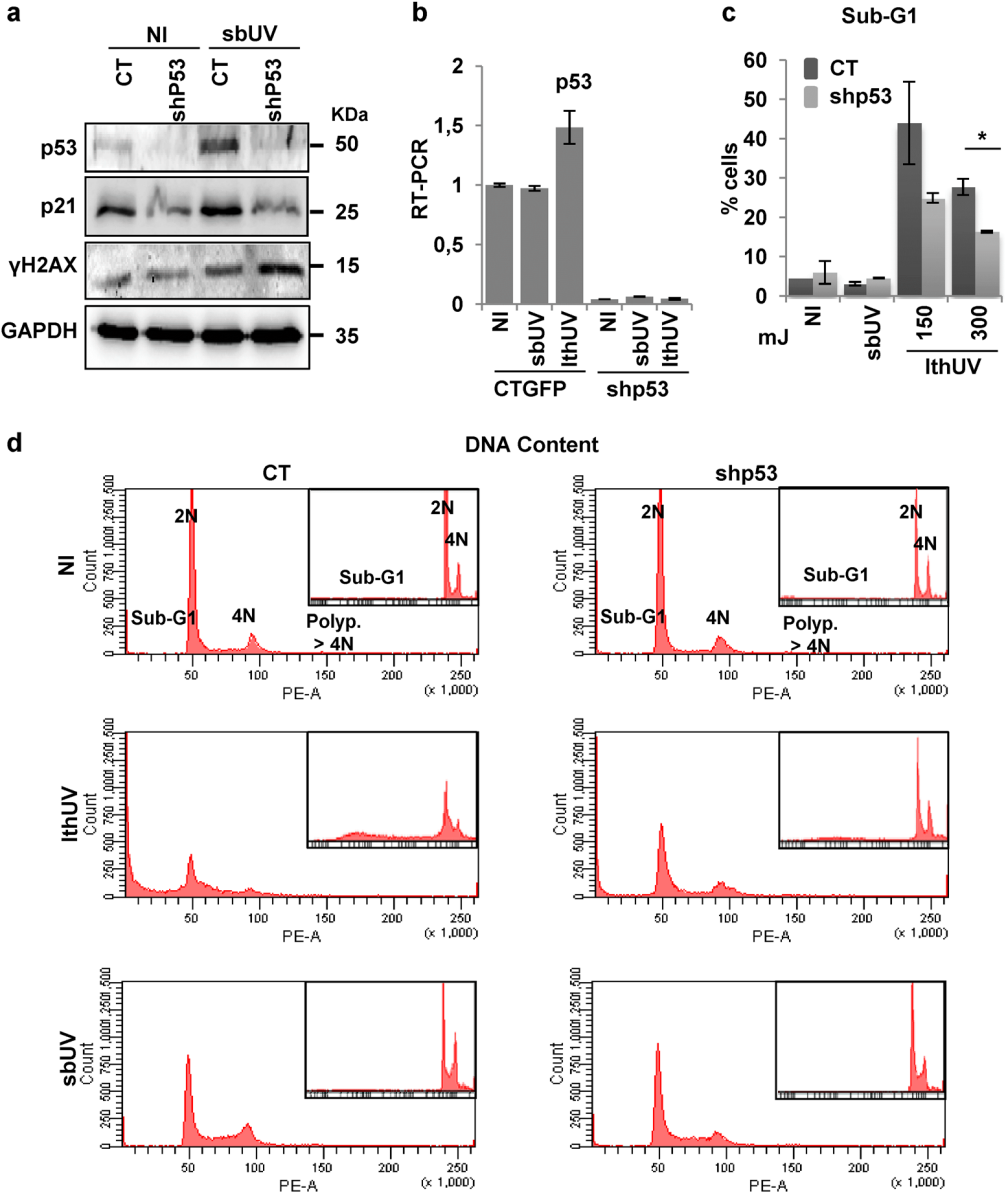
p53 mediates UV-induced keratinocyte apoptosis. Primary human keratinocytes 6 days after infections with the empty vector (CT) or with shRNA specific to p53 (shp53) and 24 h after lethal UV (150–300 mJ/cm^2^; lthUV) or sublethal UV doses (sbUV), as indicated. **a** Expression of p53, its target p21 or γH2AX by western blotting 24 h after sbUV. GAPDH as loading control. **b** Expression of p53 mRNA as measured by RT-qPCR. **c** Percent of cells in the sub-G1 (apoptotic) fraction of the cell cycle. **d** Representative flow-cytometry analyses for DNA content as in Fig. 1. Inset histograms show DNA content in logarithmic units. NI: non-irradiated. Irradiation units: /cm^2^. *p < 0.05. Data are representative or mean ± s.e.m. of triplicate samples

It has been shown that tumour suppressor p53 mediates UV-induced apoptosis in epidermal keratinocytes^5^. We tested whether p53 mediates UV-induced differentiation. We irradiated human primary control or p53 knock-down keratinocytes at lethal doses (lthUV, 150-300mJ/cm^2^) or sbUV doses of UV. p53 protein and mRNA were suppressed by exogenous expression of well established specific shRNA^12^, even upon UV (Fig. 4a, b). p53 transcriptional target, the cell cycle inhibitor p21CIP (p21), was decreased by shp53 although a significant expression was still detected (Fig. 4a). p21 has a known p53-independent expression pathway^23^. The absence of p53 provoked a higher impact of UV on keratinocyte DNA damage (γH2AX). As shown in Fig. 4c, d, lthUV irradiation induced acute apoptosis by 24 h resulting in over 40% of cells in the sub-G1 fraction. The proportion of apoptotic cells significantly decreased in the absence of p53, indicating that the tumour suppressor mediates UV-induced apoptosis. In contrast, a scarce effect was observed in the cell cycle of keratinocytes by sbUV irradiation.

**Fig. 5:**
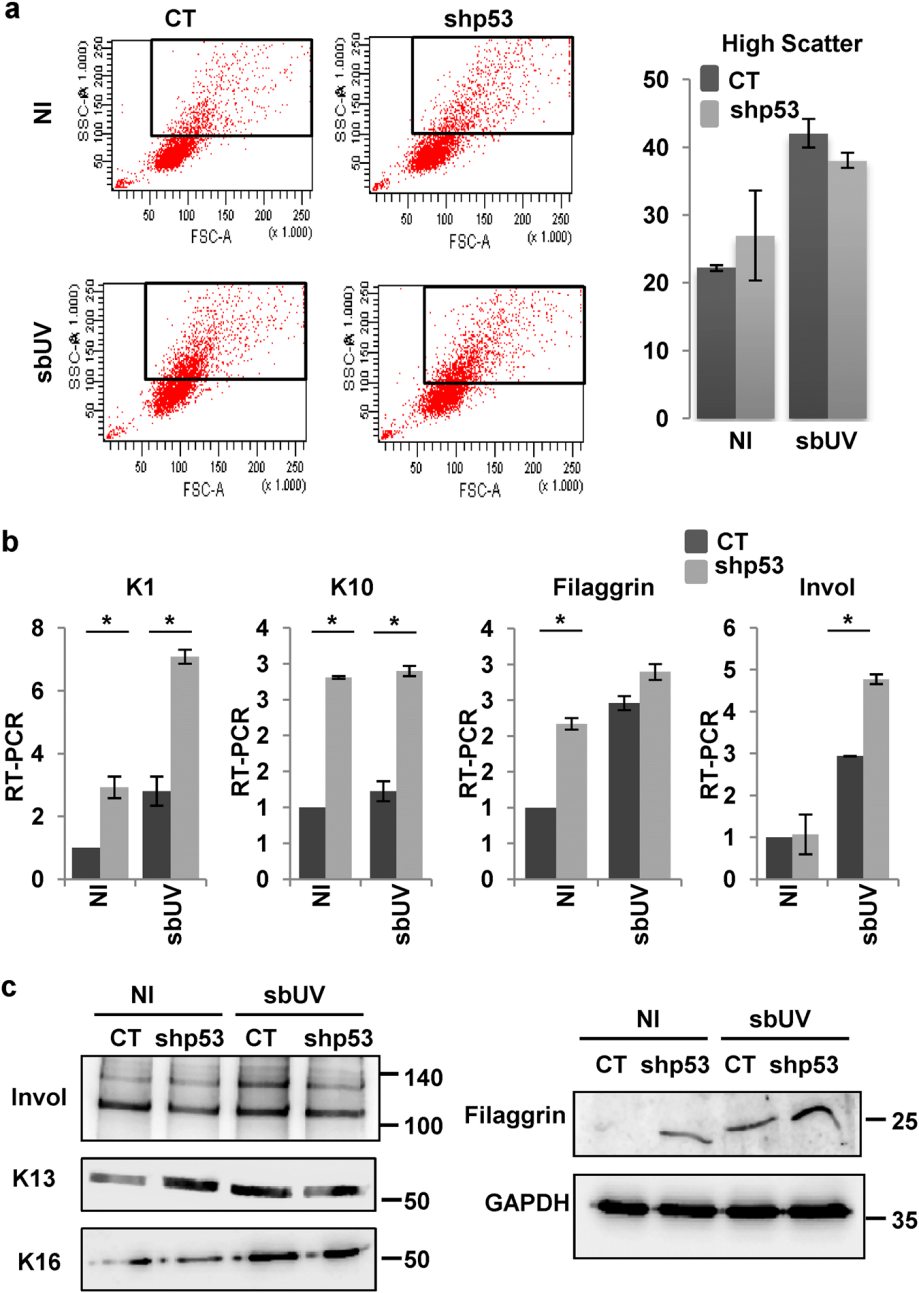
UV-induced squamous differentiation is independent of p53. Primary human keratinocytes analysed 9 days after infections with the empty vector (CT) or with shRNA specific to p53 (shp53) 24 h (**b**, **c**) or 48 h (**a**) after a sbUV dose. **a** Representative flow cytometry light scatter analyses. Black boxes gate cells with high light scatter. Bar histogram displays the percent of cells with high scatter as indicated. **b** Expression of squamous differentiation markers keratin K1, keratin K10, filaggrin or involucrin (Invol) as measured by RT-qPCR 24 h after irradiation. **c** Western blotting of cells as in (**b**) on insoluble (Invol, K13 and K16; same number of cells per lane) or soluble (Filaggrin and GAPDH as its loading control) cellular protein fractions. NI: non-irradiated. *p < 0.05. Data are representative or mean ± s.e.m. of duplicate samples

We investigated whether p53 mediated the differentiation response to sbUV levels. The inactivation of p53 alone induces squamous differentiation via cell cycle stress^12^. Contrary to the apoptosis response induced by lthUV, the loss of endogenous p53 did not compromise the induction of terminal differentiation provoked by sbUV irradiation, as measured by increased light scattering (Fig. 5a) and expression of suprabasal squamous markers (mRNA or protein) keratins K1, K10, K16 and K13, involucrin and filaggrin (Fig. 5b, c). The results show that endogenous p53 does not mediate UV-induced differentiation.

**Fig. 6:**
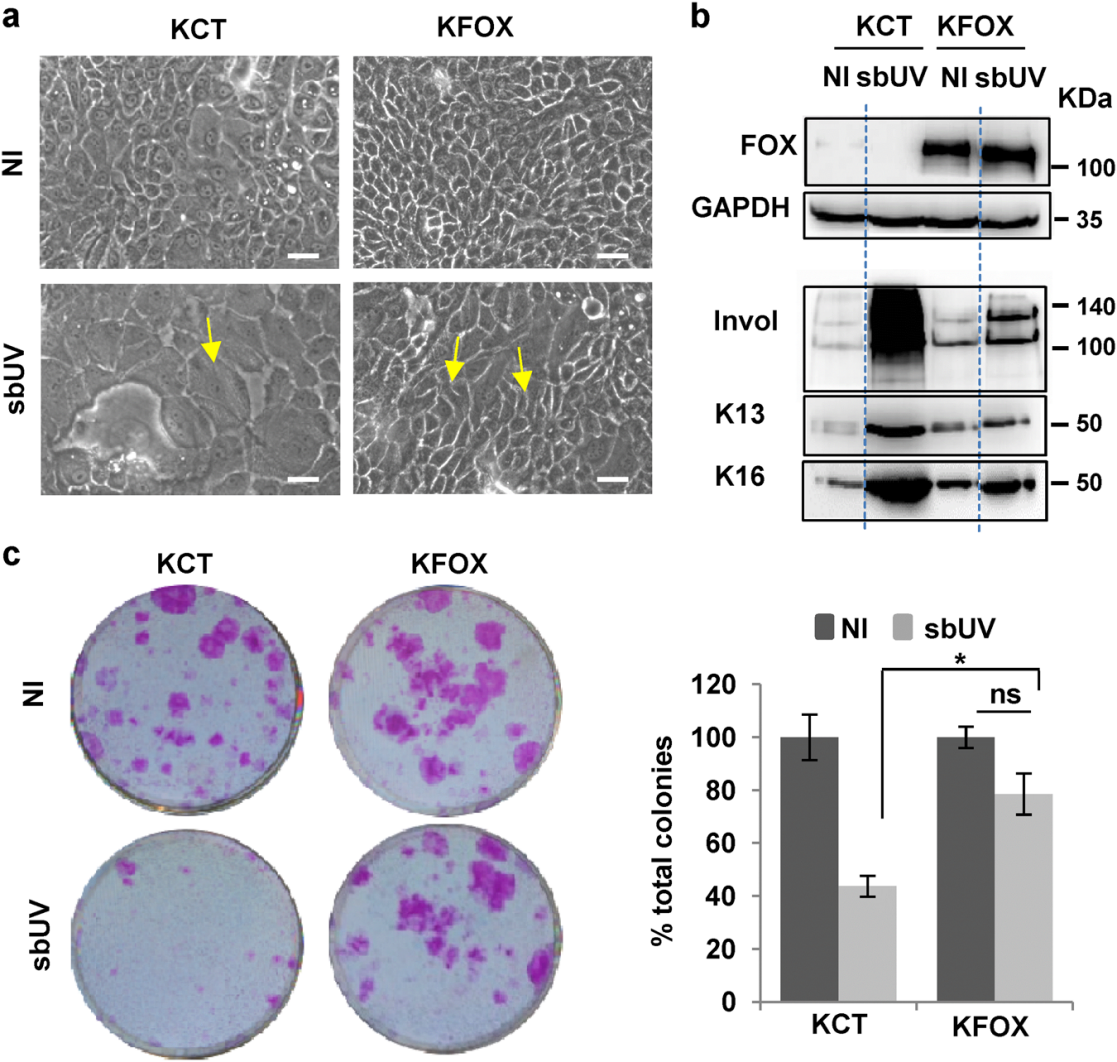
Mitotic regulator FOXM1 suppresses UV-induced squamous differentiation and rescues the clonogenic potential. Analyses of primary human keratinocytes expressing the empty vector (KCT) or the same vector expressing FOXM1 (KFOX), non-irradiated or 48 h after sbUV irradiation. **a** Representative microphotographs of phase contrast. Note that typical differentiated large cells are induced by UV whereas small proliferative cells are recovered by FOXM1 in spite of UV (yellow arrows). **b** Western blotting of KCT or KFOX on soluble (FOXM1 and GAPDH as its loading control) or insoluble (Invol, keratins K13 and K16) cellular protein fraction. **c** Clonogenic capacity of the cells indicated plated 48 h after sbUV irradiation (2500 cells per well in triplicates). Bar histogram shows the percent of colonies relative to CT 48 h after irradiation. NI: non-irradiated.ns: not statistically significant. *p < 0.05. Data are representative or mean ± s.e.m. of triplicate samples. See also Supplementary Figure 7

To test whether UV irradiation might induce differentiation via mitosis control, we overexpressed the mitosis global regulator FOXM1^24,25^. This gene allows keratinocytes to bypass the mitotic checkpoints and forces cell division^26,27^. Interestingly, overexpression of FOXM1 recovered the proliferative cell morphology (Fig. 6a) and mitigated the induction of squamous differentiation and thereby the loss of clonogenic capacity (Fig. 6b, c and Supplementary Figure 7a, b) caused by sbUV irradiation. These results suggest that mitotic checkpoints control UV-induced differentiation.

**Fig. 7:**
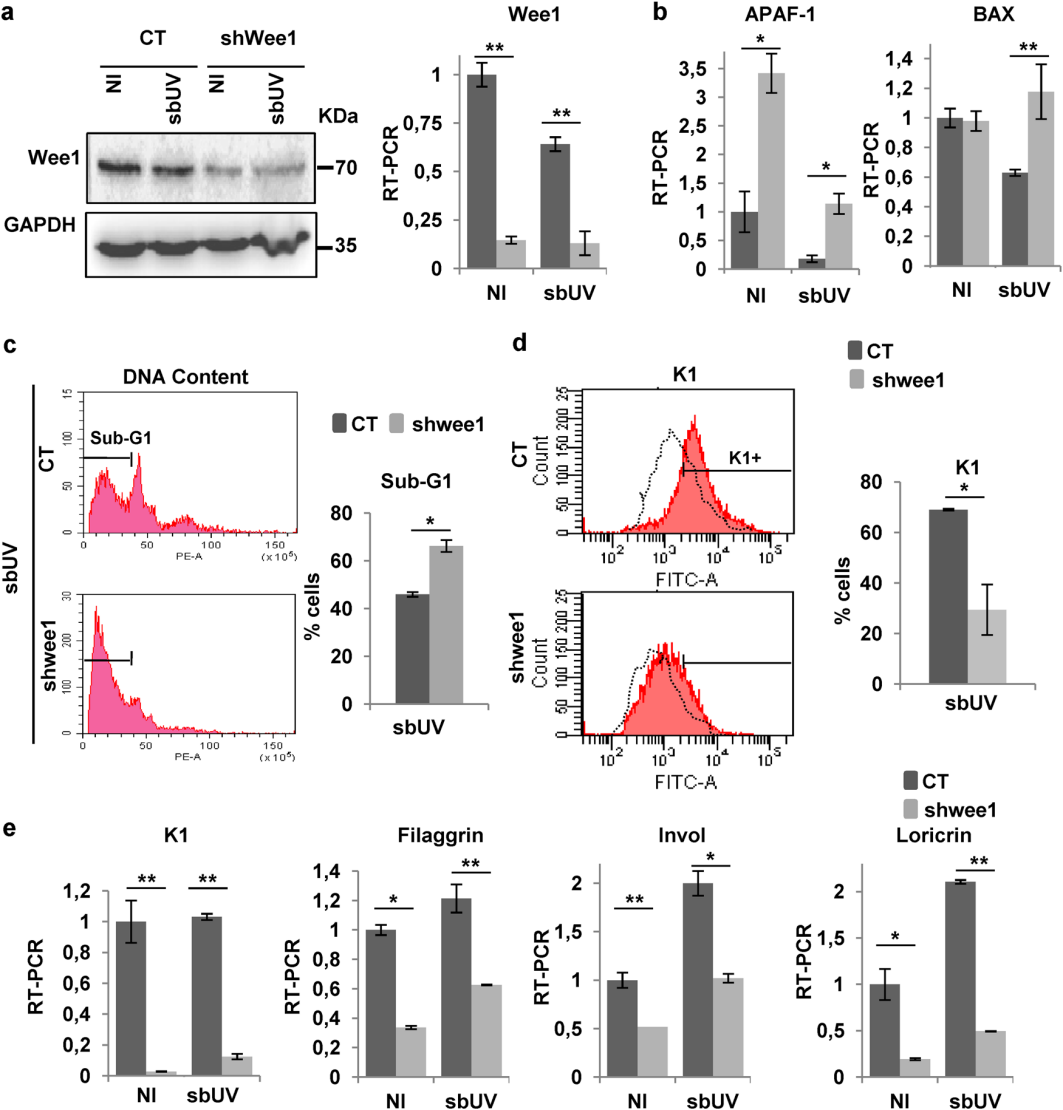
Mitosis checkpoint protein Wee1 protects keratinocytes from UV-induced apoptosis and allows UV-induced squamous differentiation. Analyses of primary human keratinocytes 3 days after infections with the empty vector (CT) or specific shRNA to Wee1 (shwee1) and 24 h (**a**), 16 h (**b**, **e**), or 72 h (**c**, **d**) after sbUV irradiation as indicated. **a** Expression of Wee1 by western blotting or by RT-qPCR. GAPDH as loading control. **b** Expression of pro-apoptotic markers APAF-1 or BAX by RT-qPCR. **c**, **d** Analyses of shedding cells collected from the culture supernatant. **c** Representative flow-cytometry of DNA content. Bar histogram shows the percent of apoptotic cells in the sub-G1 fraction. **d** Representative flow-cytometry for the expression of keratin K1. Bar histogram displays positive cells relative to NI control (black broken line). **e** Expression of differentiation markers K1, filaggrin, involucrin (Invol) or loricrin by RT-qPCR. NI: non-irradiated. *p < 0.05, **p < 0.01. Data are representative or mean ± s.e.m. of triplicate samples. See also Supplementary Figure 7

In order to confirm a role of G2/M checkpoints in the keratinocyte differentiation response to UV, we studied the involvement of Wee1 kinase. Wee1 via cdc25 inactivates the main mitotic kinase cdk1 in response to DNA damage and causes G2 arrest to allow DNA repair independently of p53^28^. We therefore knocked-down Wee1 in primary keratinocytes and subjected them to UV irradiation. We did this in low-calcium (<0.01 mM) conditions where viral infections are more efficient on primary keratinocytes (see Materials and methods) and where differentiating cells early detach into the medium^12,29–31^. Partial silencing of Wee1 (Fig. 7a) in non-irradiated cells slightly promoted proliferation without any detectable sub-G1 population in the attached monolayer (Supplementary Figure 7c, d). However, loss of Wee1 significantly resulted in apoptosis upon sbUV irradiation as monitored by increased expression of mRNA for pro-apoptotic factors BAX and APAF-1 (Fig. 7b) and increased proportion of detached apoptotic cells in sub-G1 (Fig. 7c). The inhibition of Wee1 caused sbUV-induced apoptosis at the expense of terminal differentiation according to the decrease of differentiation markers keratin K1, filaggrin, involucrin and loricrin (Fig. 7d, e).

## Discussion

The main genomic insult to the skin is UV irradiation. It is instrumental to skin homeostasis that daily, non-lethal levels of UV, trigger a squamous differentiation response. This might constitute a self-protective mechanism of the skin alternative to apoptosis, in order to suppress pre-cancerous cells via shedding (*peeling*).

UV light-induced skin squamous cancer cannot be originated by cells undergoing apoptosis but by cells surviving irradiation. Given that skin cancer is mainly due to repeated or chronic exposure to UV^1,32,33^, pre-cancerous cells are expected to arise from sublethal levels. Our results provide a mechanism by which the skin can continuously be cleansed of damaged, potentially pre-cancerous cells. Although most studies have addressed keratinocyte apoptosis induced by UV, some results have been previously reported on differentiation effects. Cotton and Spandau suggested that at sublethal UV doses differentiation might protect keratinocytes from apoptosis^34^. Increased production of cornified envelopes or expression of differentiation markers upon UV have been detected in human keratinocytes in gene arrays, ex vivo, or in organ cultures^29,35–37^. Repeated irradiation cycles triggered an alternative differentiation response in telomerase-immortalised keratinocytes^38^. Our results strongly support a physiological differentiation response to sublethal UV irradiation. We have investigated the mechanisms triggering this response.

p53 has a well-known capacity to trigger apoptosis when the cellular damage is too acute to be repaired^7^. Although the role of p53 in mediating UV-induced apoptosis is well established^4,5^, in our study the lack of p53 did not impair the differentiation response to sublethal UV irradiation. This result is consistent with the known function of p53 in holding the cell cycle to allow DNA repair^7^. In addition, we have previously shown that p53 does not mediate keratinocyte differentiation induced in response to oncogenic cell cycle stress^11,12^.

Our hypothesis is that moderate sublethal UV irradiation induces keratinocyte differentiation via the DNA damage response pathways, through the G2/M checkpoints. This hypothesis is well supported by the proliferative rescue by FOXM1 upon UV. FOXM1 is frequently amplified in epithelial cancer^39^ and is a global mitotic regulator with the capacity to override G2-mitotic checkpoints^25,40^. This transcription factor induces a set of genes driving mitosis. Altogether, the results suggest that the induction of differentiation upon UV irradiation is dependent on mitosis impairment. This is further illustrated by the requirement of Wee1.

Wee1 is a component of the DNA damage mitosis checkpoints^28^. Its role is to inactivate the central mitosis kinase cdk1 in order to delay entry in mitosis upon DNA damage. It is interesting that keratinocytes undergo mitotic slippage in the onset of differentiation^14,41^. Mitotic slippage is caused by the incapability of cells to maintain a G2 arrest^42^. It has been shown that mitotic slippage occurs when cdk1 is prematurely inactivated during a G2/M arrest^43,44^. In addition, in various systems mitotic slippage has been proposed to protect cells from apoptosis (refs in Gandarillas et al.^45^). Although p53 is part of the G2 checkpoint to promote DNA repair, the lack of p53, by not allowing full repair, provokes a G2/M arrest^12^. This can be achieved by the mitosis checkpoints but some cell cycle inhibitors such as p21 can be induced independently of p53 at G2 and thus inhibit cdk1 and cause G2/M arrest^23^. Certainly, we found a significant expression of p21 in the absence of p53 before and after UV irradiation. Wee1 also promotes cdk1 inhibition in response to DNA damage independent of p53. The fact that inactivation of Wee1 induces apoptosis indicates that this protein is involved (possibly required) in the squamous differentiation pathway.

The loss of Wee1 reduced the threshold of UV levels at which keratinocytes underwent apoptosis. This suggests that mitotic checkpoints determine the keratinocyte fate towards apoptosis or differentiation. Interestingly, moderate UV irradiation has been shown to trigger a postreplication checkpoint in yeast, an alternative to the G1/S and G2/S checkpoints triggered by high doses^46^.

The results have implications into disease. The UV-induced DMC may contribute to the therapeutic effects of moderate exposure to the Sun or UV on hyperproliferation indicated for the common skin disorder, psoriasis (4% of the population^47^). It also contributes to explaining why skin carcinomas arise mainly upon chronic sun exposure. Sustained UV irradiation promotes the expansion of p53 mutant cells^5,48^. It has been proposed that this occurs because p53 mutant cells are more resistant to UV-induced apoptosis than adjacent wild-type cells^49^. Additional alterations in the DMC caused by sustained UV irradiation might also allow p53 deficient cells to amplify with tumorigenic consequences and genomic instability.

Lastly, keratinocytes of oral mucosa and larynx appear to share with keratinocytes of the skin the same differentiation response to sublethal UV irradiation. Most epithelia of head and neck are stratified and squamous. They are not mostly exposed to the Sun but to carcinogens of the intake, especially alcohol and tobacco, yet undergo a similar response as epidermal cells to the sbUV radiation. Interestingly, the tar carcinogen 7,12-dimethylbenzanthracene (DMBA), a more frequent mutagen in the oral cavity, also induces squamous differentiation in oral keratinocytes^21^. Therefore, the regulation that we have found in keratinocytes of the skin might apply to head and neck self-renewal stratified epithelia. Alterations of this pathway might thus be common to squamous carcinomas of these locations. Self-renewal epithelia need powerful mechanisms to minimise the continuous impact of carcinogens. The DMC might renew and cleanse these tissues through squamous differentiation and cell shedding. This mechanism might protect them from precancerous mutations and thus maintain homeostasis in spite of the constant sources of DNA damage.

## Materials and methods

### Cell culture, plasmids and viral infections

Ethical permissions for this study were requested, approved, and obtained from the Ethical Committee for Clinical Research of Cantabria Council, Spain. In all cases, human tissue material discarded after surgery was obtained with written consent presented by clinicians to the patients, and it was treated anonymously.

Primary keratinocytes were isolated from neonatal human foreskin of four individuals or from adult head and neck epithelia of two individuals (inner cheek, larynx), and cultured in the presence of 3T3 mouse fibroblast feeder layer (inactivated by mitomycin C), in Rheinwald FAD medium as described (10% foetal bovine serum and 1.2 mM Ca^+2^^50,51^). For cell suspensions, keratinocytes were harvested at subconfluence and feeder cells were carefully removed by treatment with 250μM EDTA-PBS before trypsin. However, at subconfluence very few feeder cells remain. Low cell passages (1–4) were utilised. The 3T3 mouse fibroblast cell line used as feeder layer was cultured in Dulbecco medium 10% donor calf serum.

For gene delivery in primary keratinocytes, the following lentiviral constructs driven by constitutive promoters were used: control GFP pLVTHM and a construct expressing shRNA against p53: pLVUH-shp53-GFP (shp53; a gift from Patrick Aebischer and Didier Trono^52^; Addgene plasmid 11653); control pLVX (CT-pLVX) and pLVX-FoxM1 (FOXM1; kindly provided by S. Stoll, University of Michigan, Ann Arbor, USA); control plKO1 (Sigma-Aldrich, Inc) and a construct expressing shRNA against Wee1 (shwee1).

Lentiviral production was performed by transient transfection of 293T cells as previously described^12^. Lentiviral infections were performed as described^27^. Infections of Control GFP, shp53, CT-pLVX and pLVX-FoxM1 were made in Rheinwald FAD medium. Experiments involving lentiviral construct bearing shRNA to Wee1 were performed in low Ca medium due to the higher infection efficiency. In this case, cells were transferred to low-calcium concentration (<0.1 mM) in two media: Keratinocyte Growth Medium 2 Promocell; ref. C-20111 or Defined Keratinocyte-SFM (serum-free, <0.1 mM Ca^+2^; Gibco; ref. 10744019) following the manufacturer’s instructions. Low Ca conditions allow early differentiation of human keratinocytes but not stratification. Late differentiation or apoptotic cells are shed into the medium^12,29–31^.

For clonogenicity assays, 2500 keratinocytes were grown in high-calcium FAD medium and plated per T6 well triplicates. About 14 days later, the culture was stained with Rhodanile blue as described previously^53^. This mixture dyes differently keratinocyte colonies (pink) and background feeders (blue).

### Antibodies

The following primary antibodies from Santa Cruz Biotechnology were used: anti-FOXM1 (Western Blot: WB), anti-GAPDH (FL-335, WB), anti-keratin K10 (RKSE60, immunofluorescence: IF), anti-p53 (FL-393, IF and WB), anti-keratin K16 (Flow Cytometry: FC and WB) and anti-wee1 (B11, WB). Other antibodies used were: anti-involucrin (SY5, Sigma-Aldrich, IF, FC; SY3^54^, WB), anti-BrdU (BD Biosciences, FC), anti-filaggrin (PRB-417P, COVANCE, WB), anti-γH2AX (Ser139, Millipore, FC and WB), anti-keratin K1 (Poly19052, Biolegend, IF, FC), keratin K13 (KS-1A3, NOVUS, FC) and anti-p21 (P1484, Sigma-Aldrich, WB). Keratins K16 and K13 are typical of hyperplastic skin and oral epithelium, respectively and both are expressed in suprabasal cultured keratinocytes^12,21^.

The following secondary antibodies from Jackson ImmunoResearch were used: Alexa Fluor® 488-conjugated goat anti-rabbit or anti-mouse IgG antibodies (FC and IF); Alexa Fluor® 594-conjugated goat anti-rabbit or anti-mouse IgG antibodies (IF). Other secondary antibodies used were: IRdye800-conjugated goat anti-rabbit or anti-mouse IgG antibodies (Li-Cor, WB) and HRP-conjugated goat anti-rabbit or anti-mouse IgG antibodies (Bio-Rad, WB).

### Ultraviolet irradiation

Keratinocytes were irradiated with UPV CL-1000 Series UV crosslinker at various doses. The light sources were fluorescent tubes of 312 nm shortwave ultraviolet B (UVB). Keratinocytes were plated in 60 mm dishes and irradiated in subconfluence conditions (70% of the plate). Keratinocyte medium was replaced 14 h before the irradiation. Before the irradiation, the medium was removed and added PBS tempered at 37 °C. 60 mm dishes with keratinocytes were irradiated uncovered. Once keratinocytes were irradiated, PBS was removed and fresh medium was added. Non-irradiated control cells were subjected to the same procedure except for lack of irradiation.

The same cell fate was observed at doses 150– 300 mJ/cm^2^ (apoptosis; lethal UV: lthUV) or at 10–25 mJ (differentiation; sublethal UV: sbUV). Within each group, the effects were slightly faster or more intense at the higher doses. The doses were chosen depending on the timing of the assay or if the culture was high calcium or low calcium (where the non-stratified monolayer was more sensitive). The effect of sbUV doses on squamous differentiation was confirmed on primary cells from the healthy skin of four different individuals and healthy head and neck epithelia of two different individuals, with consistent results.

### Flow cytometry

Keratinocytes were harvested, fixed and stained for BrdU, DNA content (Propidium Iodide, PI), γH2AX, involucrin and keratins K16, K1 as previously described^11,12^. Keratin K13 was labelled as K1. All antibody stainings were controlled by the use of similar concentration of isotype negative antibody (mouse IgG, Sigma-Aldrich; rabbit serum, or anti-BrdU in non-BrdU containing cells). After staining, cells were firmly resuspended and filtered through a 70 µM mesh to minimise the presence of aggregates and then analysed on a Becton Dickinson FACS Canto™ and CytoFLEX (Beckman Coulter). 10,000 events were gated and acquired. For DNA synthesis analyses, cells were cultured in the presence of 10 μM BrdU (Sigma-Aldrich) for 1.5 h and harvested. For BrdU pulse-chase experiment, cells were cultured for 1.5 h in the presence of 10 μM BrdU just before irradiation (Sigma-Aldrich) and were harvested 48 or 72 h after irradiation. BrdU staining and DNA content analysis with PI (25 μg/ml, 12 h) were performed as described^11^.

### Immunodetection

For immunofluorescence, keratinocytes were grown on glass coverslips, fixed and stained as previously described^11^. For western blotting, cells were washed with PBS, lysed and subjected to SDS-PAGE electrophoresis and blotting as previously described^11^ (soluble protein fraction). An equal amount of protein was loaded onto the gel. For protein detection in the highly insoluble cellular fraction (Keratins K13, K16, involucrin), the remaining pellet after lyses was incubated in urea lysis buffer (10 mM Tris pH 8, 5% SDS, 5% β-mercaptoethanol, 4 M urea^55^). Since protein cannot be quantitated in this lysis buffer, the same number of cells were loaded onto the gel (8000 cells per lane).

### Reverse transcription and polymerase chain reaction (RT-PCR)

Total RNA was isolated and reverse-transcribed using NucleoSpin® RNA (Macherey-Nagel) and the iScript™ cDNA synthesis kit (Bio-Rad) according to the manufacturer’s instructions. The cDNAs (2 μl) were amplified by real-time PCR (Bio-Rad iQ™ SYBR Green supermix) and normalised to β-actin mRNA levels. Primers utilised in this study for human genes were: β-actin (5′-AAAATCTGGCACCACACCTTC-3′ and 5′-AGCACAGCCTGGATAGCAA-3′), APAF-1 (5′-CCTGTTGTCTCTTCTTCCAGTGT-3′ and 5′-CTGAAACCCAATGCACTCCC-3′), BAX (5′-TGGAGCTGCAGAGGATGATTG-3′ and 5′-CCAGTTGAAGTTGCCGTCAGA-3′), filaggrin (5′-GGCACTGAAAGGCAAAAAGG-3′ and 5′-AGCTGCCATGTCTCCAAACTA-3′), involucrin (5′-TGCCTGAGCAAGAATGTGAG-3′ and 5′-AGCTGCTGATCCCTTTGTGT-3′), keratin K1 (5′-CCAGCCAGAGTAGGACCAGT-′3 and 5′-TGCAGCAAAACAAGGAAATG-′3), keratin K10 (5′-AATGAAAAAGTAACCATGCAGAATCTG-′3 and 5′-CACGAGGCTCCCCCTGAT-′3), loricrin (5′ TCATGA TGCTACCCGAGGTTTG-′3 and 5′-CAGAACTAGATGCAGCCGGAGA-′3), p53 (5′-GTTCCGAGAGCTGAATGAGG-′3 and 5′-TCTGAGTCAGGCCCTTCTGT-′3) and wee1 (5′-GCTTGCCCTCACAGTGGTAT-3′ and 5′-GTAAGGCTGACAGAGCGGTT-3′).

### Senescence

The expression of senescence marker β-Galactosidase^22^ was analysed by Senescence Cells Histochemical Staining Kit (Sigma-Aldrich) following the manufacturer's instructions and visualised and photographed by bright field microscopy (ECLIPSE TS100F and LEDCMOS 5MP COLOR Nikon).

### Statistical analyses

Data sets were compared using an unpaired Student’s *t* test. A *p*-value ≤0.05 was considered statistically significant.

## Electronic supplementary material

Supplementary Figures
