# Supplementary material for: Sublethal UV irradiation induces squamous differentiation via a p53-independent, DNA damage-mitosis checkpoint
Source: Cell Death Dis. 2018 Oct 25;9(11):1094. doi: 10.1038/s41419-018-1130-8 (PMC6202398; doi:10.1038/s41419-018-1130-8)
Supplement: Supplementary file 1 — Supplementary Figures [file 41419_2018_1130_MOESM1_ESM.pdf]

SUPPLEMENTARY FIGURES

Sublethal UV irradiation induces squamous differentiation via a p53-independent, DNA damage-mitosis checkpoint.

Isabel de Pedro, Pilar Alonso-Lecue, Natalia Sanz-Gómez, Ana Freije and Alberto Gandarillas.

de Pedro et al
Supplementary Figure 1

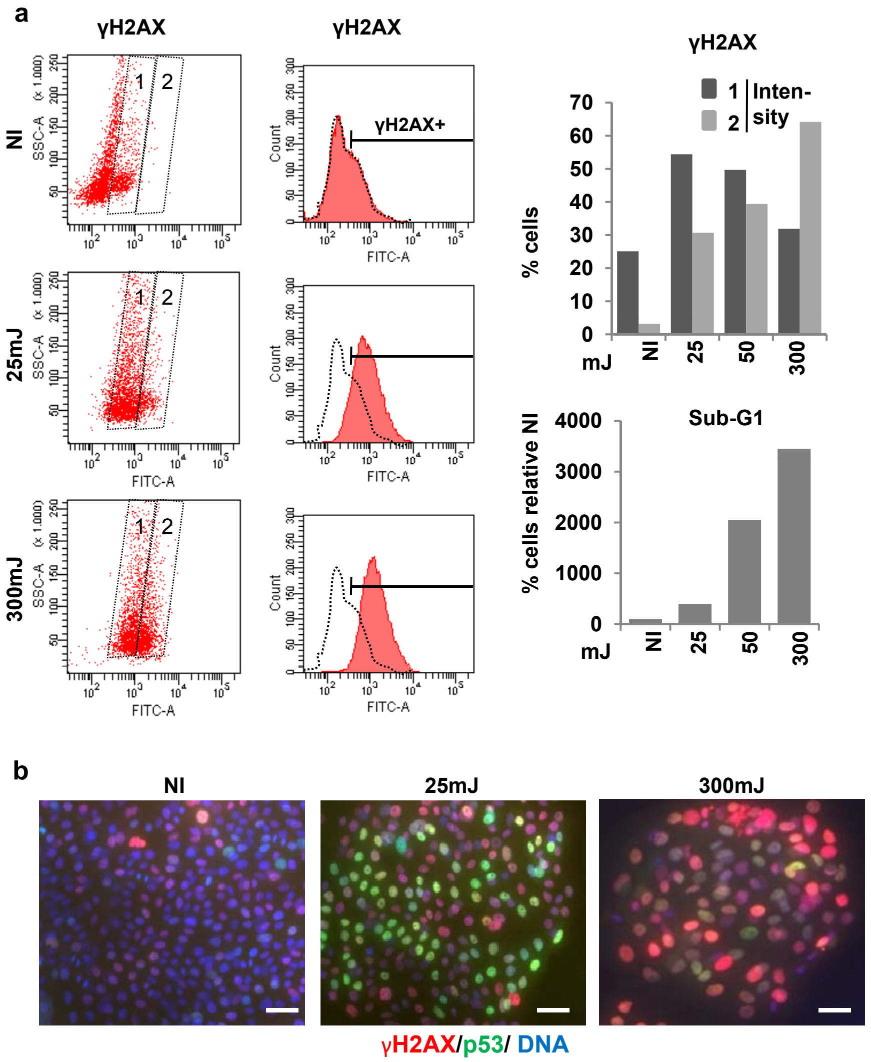

Supplementary Figure 1. Increasing doses of UV irradiation early induce increasing DNA damage and apoptosis in epidermal keratinocytes.

Primary human keratinocytes were exposed to increasing doses of UV (25-300 mJ/cm^2) and analysed 5 h after irradiation as indicated.

a. Left panels, representative dot plots (black boxes gate cells according to the 2 levels of intensity (1-2) and histograms (positive cells according to NI, black broken line) for the expression of the γH2AX (DNA damage marker). Upper right, bar histogram displays the percent of γH2AX positive cells. Lower right, bar histogram shows the percent of apoptotic cells (Sub-G1) relative to control (NI). **b.** Double immunofluorescence for γH2AX (red) and p53 (green) as indicated. DAPI for DNA in blue. NI: Non-irradiated. Irradiation units: /cm^2. Scale Bar: 50 µm.

de Pedro et al
Supplementary Figure 2

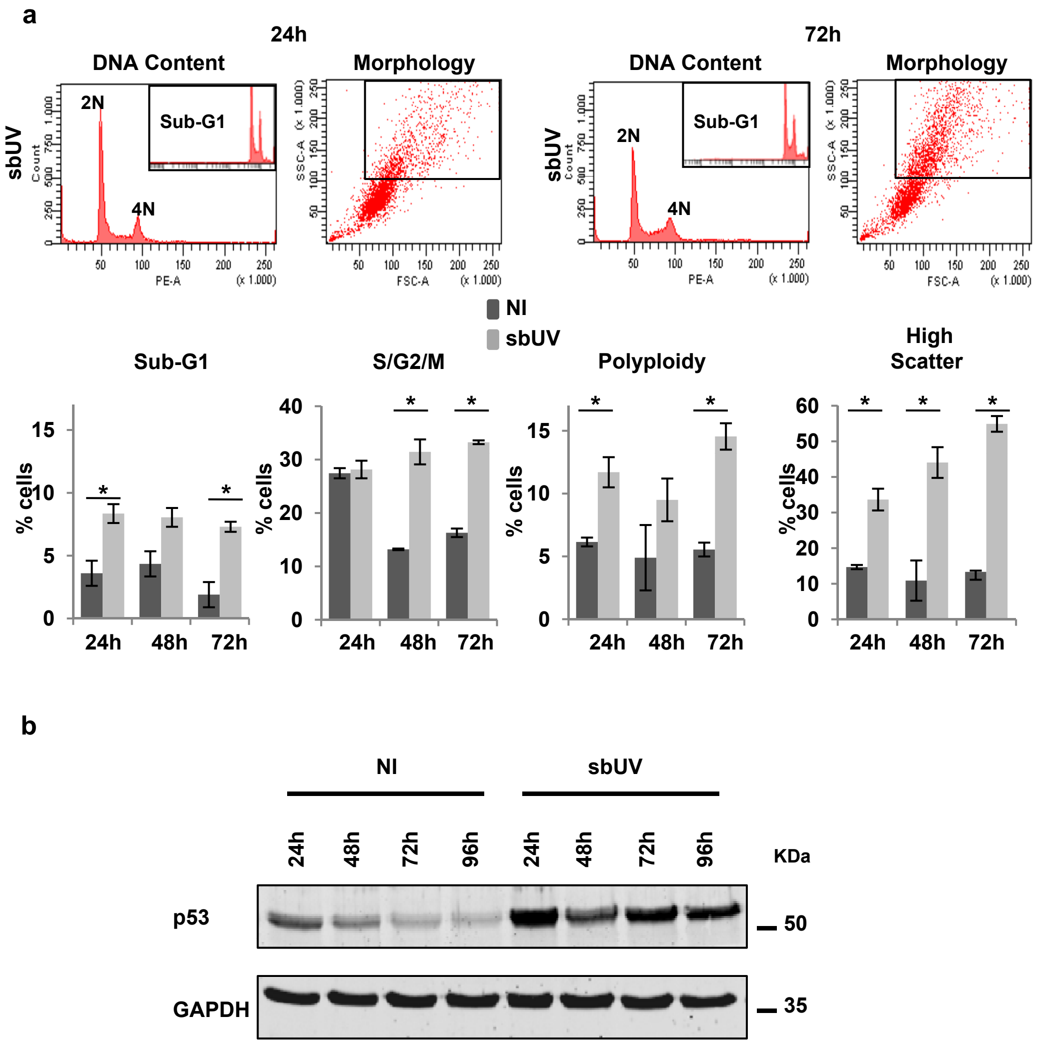

Supplementary Figure 2. Sublethal UV irradiation induces polyploidisation and high light scattering in epidermal keratinocytes.
Primary human keratinocytes 24, 48, or 72 h after sbUV irradiation as indicated.
a. Representative flow cytometry analyses for DNA content and morphology (light scatter). Logarithmic insets show the Sub-G1. Black boxes gate cells with high light scatter. Bar histograms: quantitations of the Sub-G1, S/G2/M and polyploid (<4N) fractions of the cell cycle and high light scatter (differentiated morphology). **b.** Expression of p53 by western blotting. GAPDH as loading control. NI: Non-irradiated. *p<0.05. Data are representative or mean±s.e.m. of triplicate samples. It complements Figure 1.

de Pedro et al
Supplementary Figure 3

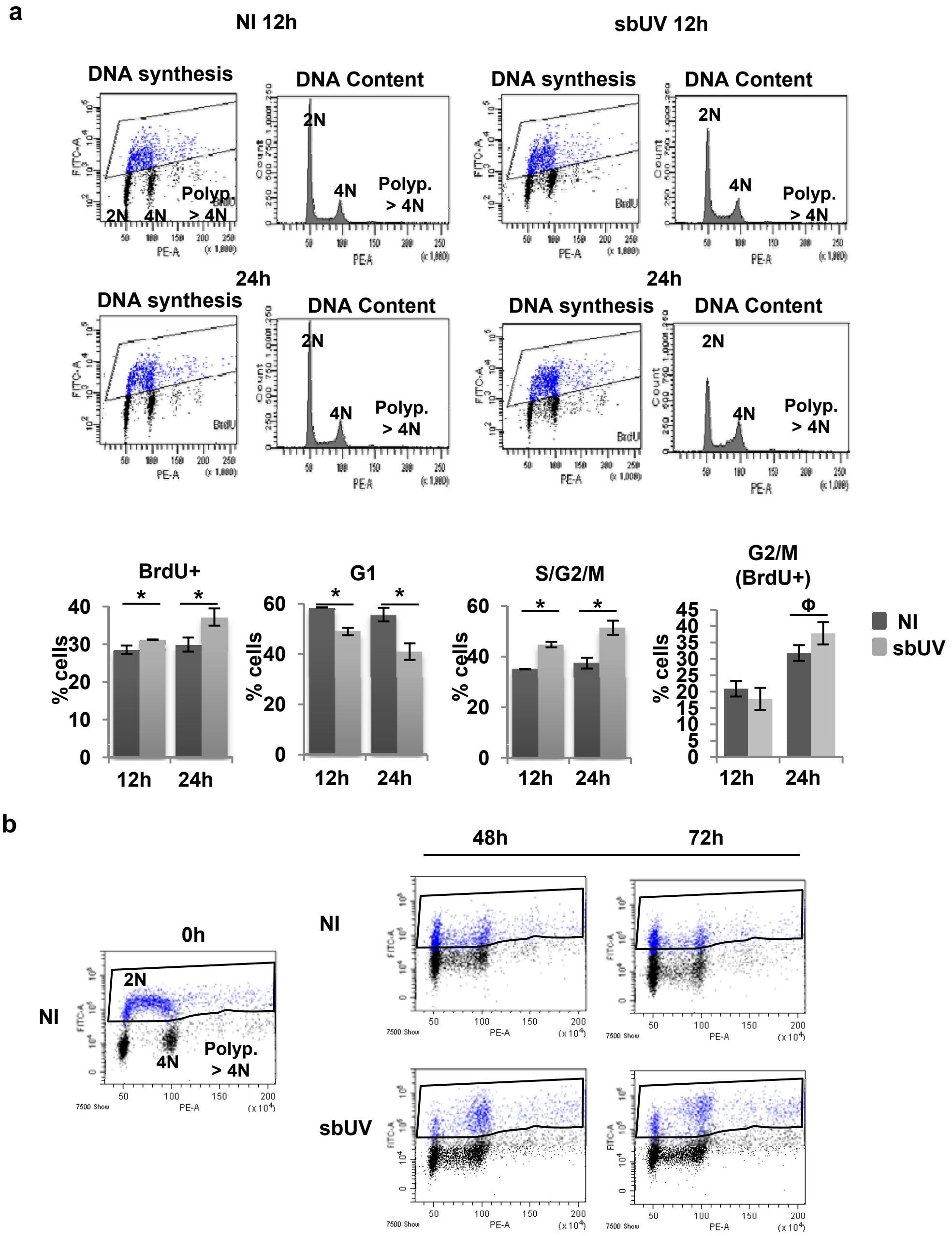

Supplementary Figure 3. Sublethal UV irradiation induces DNA synthesis, G2/M arrest and re-replication in epidermal keratinocytes. Primary human keratinocytes 12 h or 24 h (a), 48 h or 72 h (b) after sbUV irradiation. **a**. Representative flow cytometry dot plot analyses of DNA synthesis as measured by BrdU incorporation (positive cells in blue) vs DNA content. Histograms show DNA content as indicated. Bar histograms from left to right display the percent of: total BrdU positive cells; total cells in G1; total cells in S/G2/M; BrdU positive cells in G2/M. **b**. Representative flow-cytometry dot plot analyses of BrdU positive cells labeled during 1.5 h just before irradiation (pulse), or 48 or 72 h after irradiation (chase; positive cells in blue). Polyp.: polyploidy. NI: Non-irradiated. * $p<0.05$, $^{\Phi}$ $p=0.06$. Data are representative or mean±s.e.m. of triplicate samples. It complements Figure 1.

de Pedro et al
Supplementary Figure 4

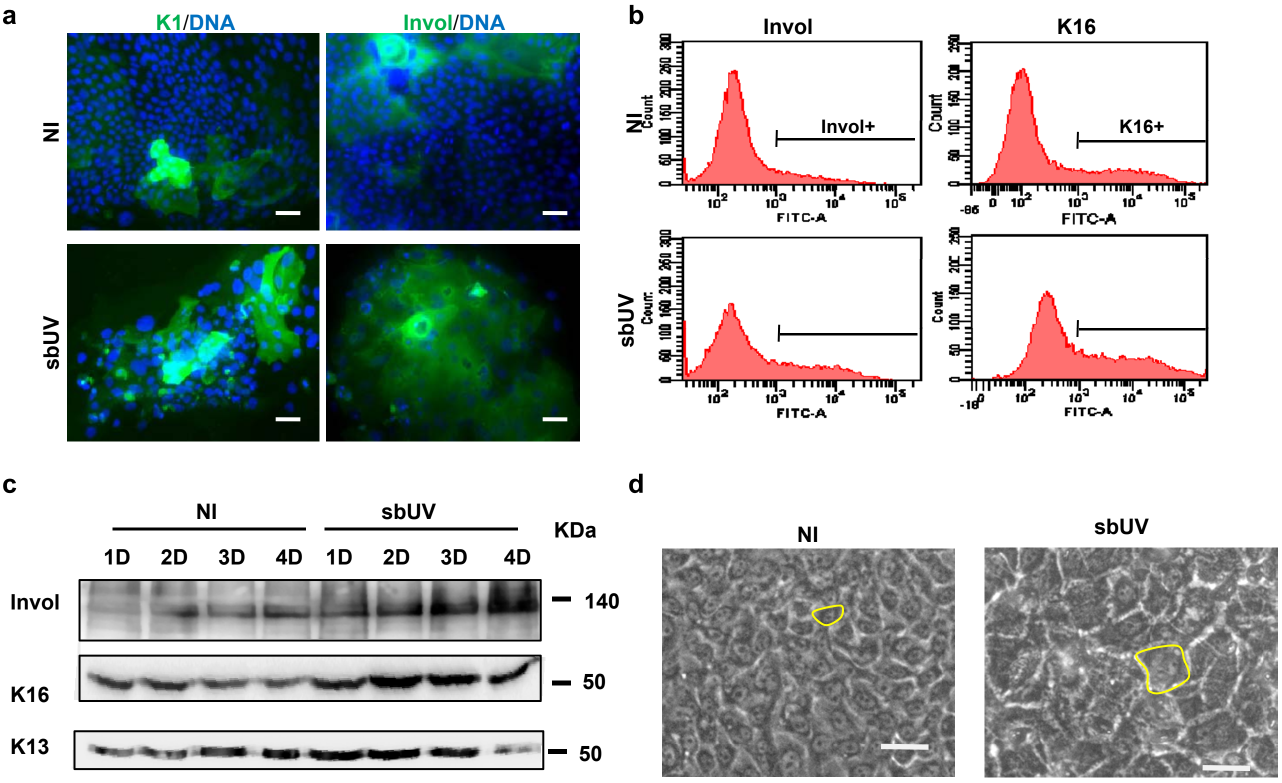

Supplementary Figure 4. Sublethal UV irradiation induces squamous differentiation in epidermal keratinocytes.
a. Representative microphotographs of immunofluorescence for keratin K1 (green, left panel) or involucrin (Invol; green, right panel) 48 h after sbUV irradiation. DAPI for DNA in blue. **b.** Representative flow cytometry analyses for the expression of Invol or keratin K16 (K16) 48 h after sbUV irradiation. **c.** Time course of the expression of differentiation markers by western blotting on insoluble cellular protein fractions (invol, K16 and K13; same number of cells per lane). **d.** Representative microphotographs of phase contrast 24 h after sbUV irradiation. Note the large size of irradiated cells typical of terminal differentiation in contrast to small proliferative NI cells (yellow lines). NI: non-irradiated. D: days. Scale Bar: 50 µm. Data are representative or mean±s.e.m. of triplicate samples. It complements Figure 2.

.

de Pedro et al
Supplementary Figure 5

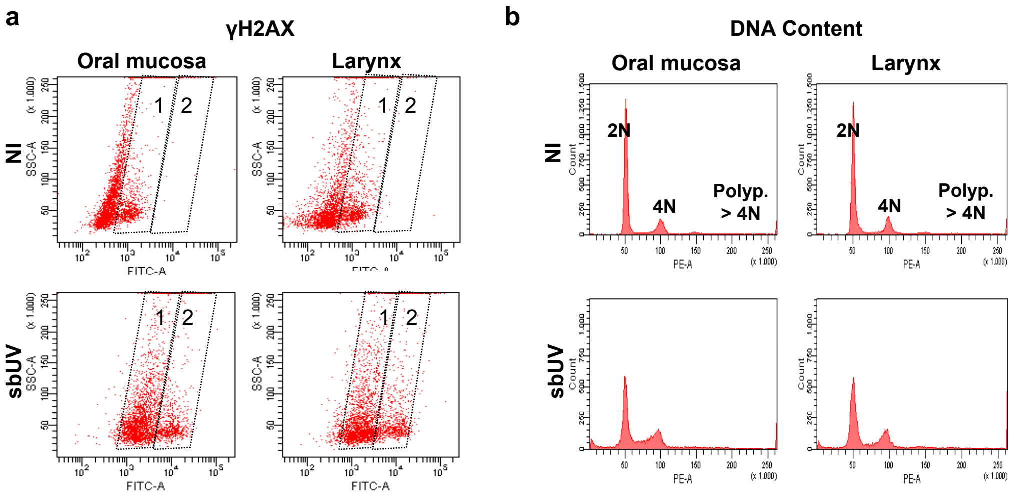

Supplementary Figure 5. Sublethal UV irradiation induces DNA damage and S phase accumulation in keratinocytes of head and neck.

Primary cells from oral mucosa or larynx, as indicated, were exposed to sbUV and analysed 5 h (**a**) or 48 h (**b**) after irradiation. **a.** Representative flow-cytometry analyses of γH2AX (early DNA damage marker). Black boxes gate cells according to 2 levels of intensity (1-2). **b.** Representative flow-cytometry analyses of DNA content. NI: Non-irradiated. Polyp.: polyploidy. It complements Figure 3.

de Pedro et al
Supplementary Figure 6

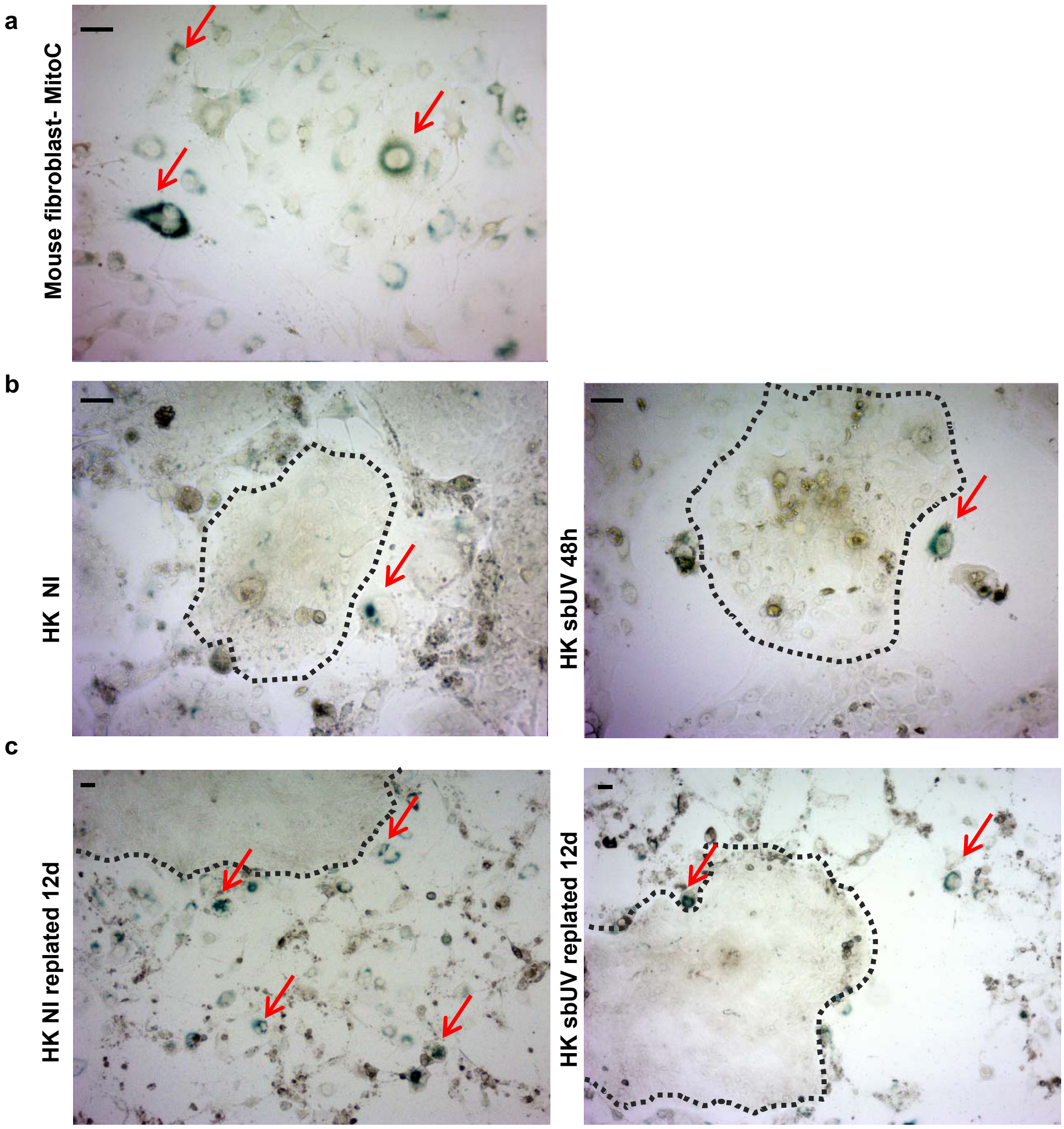

Supplementary Figure 6. Sublethal UV irradiation does not induce senescence in epidermal keratinocytes.

Representative microphotographs (clear field) of Beta-Galactosidase (β-Gal) staining to detect senescence. **a.** 3T3 mouse fibroblasts treated with mitomycin C for 2 h (400 µg/ml; MitoC). **b.** Primary human keratinocytes (HK) non-irradiated (HK NI) or 48 h after sbUV irradiation (HK sbUV 48h). **c.** Non-irradiated HK or HK that were sbUV irradiated were replated and cultured for 12 d and subjected to β-Gal staining. In b,c human keratinocytes were cultured in the presence of a fibroblast feeder layer previously treated with mitomycin C as in a. Note β-Gal positive fibroblasts in blue. β-Gal was barely detectable in the keratinocyte colonies. Black broken lines highlight human keratinocyte colonies. Scale Bar: 50 µm.

de Pedro et al
Supplementary Figure 7

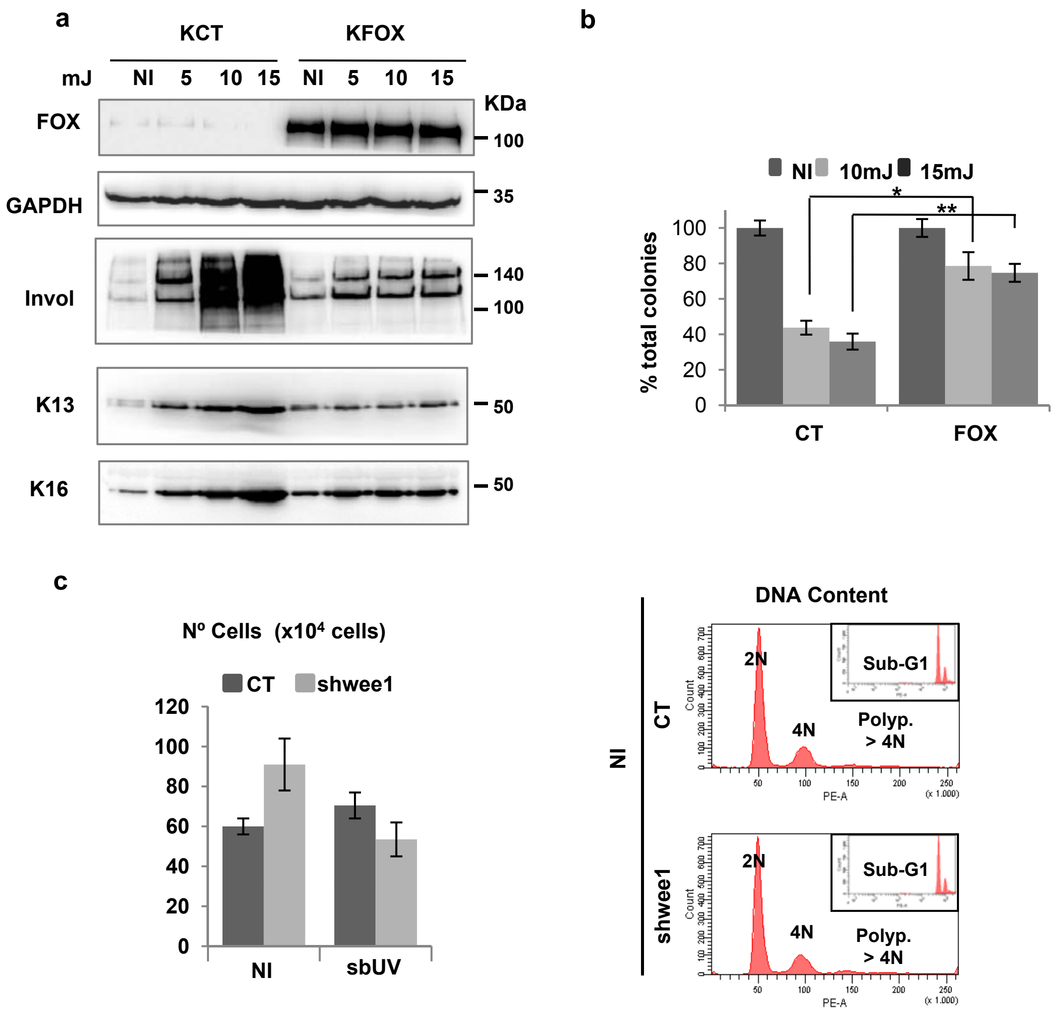

Supplementary Figure 7. FOXM1 and Wee1 in irradiated keratinocytes.
a. Uncropped blot for the proteins shown in Fig. 6b in keratinocytes expressing empty vector (KCT) or ectopic FOXM1 (KFOX). **b.** Quantitation of the number of colonies after plating non-irradiated or sbUV (10-15 mJ/cm^2.) irradiated keratinocytes corresponding to Figure 6c. **c.** Primary human keratinocytes 3 days after infections with the empty vector (CT) or specific shRNA to Wee1 (shwee1) and 24 h after sbUV irradiation as indicated. Left: bar histogram displays the number of cells. Right: representative flow-cytometry analyses of DNA content of non-irradiated (NI) cells. Polyp.: polyploidy. Irradiation units: /cm^2. It complements Figure 6 and Figure 7.
